# Supplementary material for: Patterns of Intron Gain and Loss in Fungi
Source: PLoS Biol. 2004 Nov 30;2(12):e422. doi: 10.1371/journal.pbio.0020422 (PMC532390; doi:10.1371/journal.pbio.0020422)
Supplement: Table S1 — Also available at http://genes.mit.edu/NielsenEtAl/. (4.3 MB ZIP). [file pbio.0020422.st001.zip › NielsenEtAl/html/1347.html]

AN0232.1.NCU01511.1.MG01086.1.FG04402.1


```
 CLUSTAL W (1.82) Multiple Sequence Alignments - Introns Inserted


Sequence 1: MG01086.1	277 aa
Sequence 2: FG04402.1	272 aa
Sequence 3: NCU01511.1	290 aa
Sequence 4: AN0232.1	266 aa
Alignment Length: 298 aa
Number Identitical Residues: 181 aa
Alignment Score (without introns) 7869


MG01086.1 	MAHSHSHDGSA------PHSHSHDGST---FNAQEHGHSHEILTGPGSYLGREMPIVEGR
NCU01511.1	MSHTHSHDHGDGGHHHHPHSHSHDFNSQSGFNAQEHGHSHEILDGPGSFLGREMPIVEGR
FG04402.1 	--MSHSHDG---------QSHSHDG-----FNAQEHGHSHEILDGPGSFLGREMPIVEGR
AN0232.1  	MAHSHSH------------SHSHDN-------IADHGHSHEILDGPGSYLNREQPLIEDR
          	 : :***            *****          :******** ****:*.** *::*.*

MG01086.1 	DFSERAFTIGIGG2PVGSG----K~TALMLALCLALRDTHSLAAVTNDIFTR~EDAEFLT
NCU01511.1	NWDERAFTIGIGG2PVGSG----K~TALMLNLCLALRYEHSLAAVTNDIFTR~EDAEFLT
FG04402.1 	EWSERAFTIGIGG~QDQSLSQLLT0TALMLALSLALRKEYSLAAVTNDIFTR~EDAEFLT
AN0232.1  	DWSDRAFTIGIGG2PVGSG----K~TALMLALCRALRDEYNIAAVTNDIFTR2EDAEFLT
          	::.:*********    *     . ***** *. ***  :.:********** *******

MG01086.1 	RNKALPPSRIRAIETGGCPHAAVREDISANLACLEDLHRTFNGVDLLLIESGGDNLAANY
NCU01511.1	RHKALPPQRIRAIETGGCPHAAVREDISANLAALEDLQAEFD-TDLLLIESGGDNLAANY
FG04402.1 	RNKALPPSRIRAIETGGCPHAAVREDISQNLAALEDLHREFD-TDILLIESGGDNLAANY
AN0232.1  	RNKALSPDRIRAIETGGCPHAAVREDISANLLALQNLHRQFQ-TDLLLIESGGDNLAANY
          	*:***.*.******************** ** .*::*:  *: .*:**************

MG01086.1 	SRELADFIIYVIDVSGGDKIPRKGGPGITQSDLLVVNKTDLAEIVGADLGVMERDARKMR
NCU01511.1	SRELADYIIYVIDVAGGDKVPRKGGPGITQSDLLVVNKTDLAEAVGADLAVMERDARKMR
FG04402.1 	SRELADFIIYVIDVSGGDKIPRKGGPGITQSDLLIVNKTDLAELIGADLGVMERDARKIR
AN0232.1  	SRELADFIIYVIDVAGGDKVPRKGGPGITGSDLLVVNKIDLAEAVGADISVMERDAAKMR
          	******:*******:****:********* ****:*** **** :***:.****** *:*

MG01086.1 	EGGPTVFAQVKKSVGVEHIVNLILSAWKASGAEEI---AKANGGPRPTEGLEELNA----
NCU01511.1	EGGPTVFAQVKKGVGVEHIAGLILSAWRASGAEEN---RRRKGGPRPTDGLKDLERLASP
FG04402.1 	EGGPTIFAQVKKDVGVDHIISLIKSAWKASGAEDE---RRSKGGPRPTEGLEQLE-----
AN0232.1  	EGGPTVFAVVKKDQGVDHIVNLILSAWKASGAYELSLQRWKNGAPRGSGSVEP-------
          	*****:** ***. **:** .** ***:**** : : .   :*.** : .::        

MG01086.1 	-
NCU01511.1	S
FG04402.1 	-
AN0232.1  	-
          	
```
